# Sex-Specific Role for Adenylyl Cyclase Type 7 in Alcohol Dependence

**DOI:** 10.1016/j.biopsych.2011.01.037

**Published:** 2011-06-01

**Authors:** Sylvane Desrivières, Sergey P. Pronko, Anbarasu Lourdusamy, Francesca Ducci, Paula L. Hoffman, Norbert Wodarz, Monika Ridinger, Marcella Rietschel, Diana Zelenika, Mark Lathrop, Gunter Schumann, Boris Tabakoff

**Affiliations:** a Medical Research Council Social, Genetic and Developmental Psychiatry, King's College London, United Kingdom; b Institute of Psychiatry, St. George's University of London, United Kingdom; c Department of Pharmacology, School of Medicine, University of Colorado Denver, Aurora, Colorado; d Department of Psychiatry, Psychosomatics and Psychotherapy, University of Regensburg, Regensburg, Germany; e Department of Genetic Epidemiology in Psychiatry, Central Institute of Mental Health, University of Heidelberg, Mannheim, Germany; f Center National de Genotypage, Évry, Paris

**Keywords:** *ADCY7*, adenylyl cyclase, alcohol dependence, gender, genetics, sex

## Abstract

**Background:**

Alcohol has been shown to critically modulate cyclic adenosine-3′,5′ monophosphate (cAMP) signaling. A number of downstream effectors that respond to the cAMP signals (e.g., protein kinase A, cAMP response element binding protein) have, in turn, been examined in relation to alcohol consumption. These studies did not, however, delineate the point at which the actions of alcohol on the cAMP cascade might translate into differences in drinking behavior. To further understand the role of cAMP synthesis in alcohol drinking and dependence, we investigated a specific adenylyl cyclase isoform, adenylyl cyclase (AC) Type 7, whose activity is selectively enhanced by ethanol.

**Methods:**

We measured alcohol consumption and preference in mice in which one copy of the *Adcy7* gene was disrupted (*Adcy7*^*+/−*^). To demonstrate relevance of this gene for alcohol dependence in humans, we tested the association of polymorphisms in the *ADCY7* gene with alcohol dependence in a sample of 1703 alcohol-dependent individuals and 1347 control subjects.

**Results:**

We show that *Adcy7*^*+/−*^ female mice have higher preference for alcohol than wild-type mice, whereas there is little difference in alcohol consumption or preference between *Adcy7*^*+/−*^ male mice and wild-type control subjects. In the human sample, we found that single nucleotide polymorphisms in *ADCY7* associate with alcohol dependence in women, and these markers are also associated with *ADCY7* expression (messenger RNA) levels.

**Conclusions:**

These findings implicate adenylyl cyclase Type 7 as a critical component of the molecular pathways contributing to alcohol drinking and the development of alcohol dependence.

Cyclic adenosine-3′,5′ monophosphate (cAMP) is an intracellular second messenger that mediates signaling generated by activation of numerous G protein-coupled receptors. Cyclic AMP is formed from adenosine triphosphate by the action of various isoforms of the enzyme adenylyl cyclase (AC), each of which is specifically regulated by G proteins, calcium, and other modulators (1). Within the cell, changes in cAMP levels affect the activity of protein kinase A (PKA) and subsequently the downstream targets of PKA, including cAMP response element binding protein (CREB), a transcription factor that regulates the expression of numerous genes (2).

Alcohol dependence is a psychiatric disorder that is influenced by both genetic and environmental factors (3,4). Evidence suggests (5) that there are numerous gene/proteins and gene × environment interactions that influence the degree of alcohol consumption. There is a significant amount of animal literature that describes a role of PKA and CREB in alcohol preference as well as in neuroadaptation to alcohol (i.e., alcohol dependence and tolerance) (6–8). For example, it has been reported that CREB and phosphorylated (activated) CREB are lower in the amygdaloid structures of lines of rats selectively bred for alcohol preference (P rats) and that acute exposure of these rats to alcohol increased the levels of phosphorylated CREB (9). In addition, infusion of a PKA inhibitor into the central amygdaloid nucleus of P rats decreased phosphorylated CREB levels and resulted in increased alcohol consumption (9). Infusion of a PKA inhibitor into the nucleus accumbens of Sprague–Dawley rats also increased alcohol consumption (10). Moreover, PKA mutant mice (lacking the RIIβ regulatory subunit of PKA) had lower cAMP-stimulated PKA activity in brain and displayed higher levels of voluntary alcohol consumption than wild-type (WT) mice (11). These data indicate that lower brain levels of the downstream signaling proteins of the AC-cAMP pathway might contribute to increased alcohol consumption (although one study found lower alcohol consumption in mice with low Gs α or PKA levels in brain (12).

However, surprisingly little attention has focused on the role of ACs, the family of enzymes that produce cAMP, in modulating alcohol-drinking behavior. Animal studies have shown that cAMP levels and certain isoforms of AC play a role in sensitivity to the acute sedative and ataxic effects of ethanol (13–15). In humans, it has been suggested that low platelet AC activity levels might be a genetic marker of a predisposition to alcohol dependence (16–18).

Our studies of the acute stimulatory effect of alcohol on receptor-activated AC activity showed that the AC7 isoform is the most sensitive to alcohol (19). This isoform of AC is found in human erythroleukemia cells, which are derived from platelet precursor cells (20,21) and might therefore be an important factor when platelet AC activity is measured in humans. To determine whether the ethanol-sensitive AC7 isoform influences alcohol drinking, we assessed voluntary alcohol intake and preference in mice that are genetically deficient in AC7 (22). We also evaluated genetic polymorphisms in the human AC7 gene (*ADCY7*) that could affect AC7 protein levels in a population of alcohol-dependent subjects, given that differences in AC7 expression levels and therefore activity might affect the level of alcohol drinking in humans as well as in mice.

## Methods and Materials

### Animals

Experiments were performed on 90–120-day-old mice. The *Adcy7* heterozygous “knockdown” mice, in which one copy of the *Adcy7* gene was disrupted (*Adcy7*^*+/−*^) (22), were backcrossed to C57BL/6 mice for 13–15 generations or to 129S6/SvEv mice for 13–14 generations (disruption of both copies of the *Adcy7* gene produced death in the fetus). The respective littermates were used as control subjects (WT). The animals were housed in a colony room with 12-hour light/12-hour dark cycle with controlled temperature (23°C) and humidity (40%). Access to food and water was ad libitum. All efforts were made to minimize any potential suffering, and all experimental procedures were approved by the University of Colorado Denver Institutional Animal Care and Use Committee.

### Two-Bottle Choice Ethanol Consumption

Animals from both genetic backgrounds (C57BL/6 and 129S6/SvEv) were used and tested in separate experiments. Mice were singly housed for 4 days before the experiment with continuous access to two drinking tubes containing tap water only, to reduce stress and acclimatize the animals. On the fifth day, one drinking tube was filled with a solution containing ethanol. The concentration of ethanol was increased every fourth day (3%, 5%, 7%, 10%, and 14%, v/v). Drinking tube position was changed every other day, and ethanol and water consumption were measured every day. The average ethanol consumption (g/kg/day) and the preference ratio for ethanol (ethanol volume/total fluid volume consumed) from each of the 4 day sessions was used for the analysis.

### Analysis of Mouse Alcohol Drinking Data

**Figure 1:**
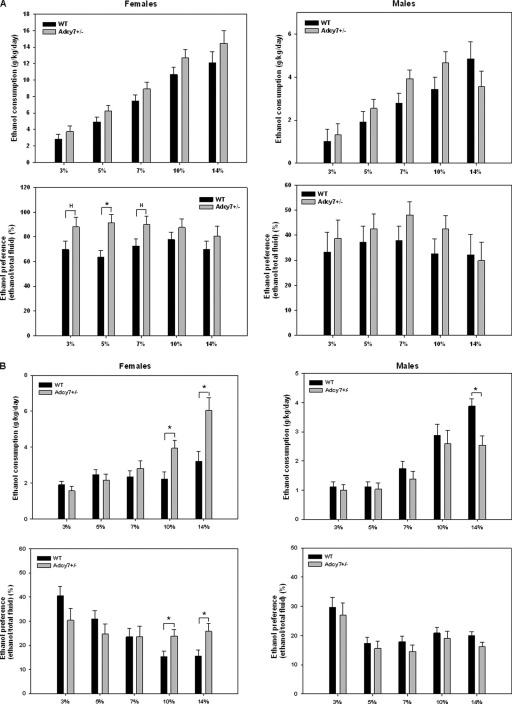
Ethanol-drinking patterns in *Adcy7*^*+/−*^ mice on the C57BL/6 **(A)** and 12956/SvEv **(B)** backgrounds. Daily ethanol consumption and preference (for 3%–14% ethanol in water solution) were measured by the two-bottle free-choice paradigm in male and female *Adcy7*^*+/−*^ and wild-type (WT) mice (Adcy7^+/−^ C57BL/6: females, *n* = 6; males, *n* = 11; WT, females *n* = 8, males *n* = 8; Adcy7^+/−^12956/SvEv: females *n* = 9, males *n* = 7; WT, females *n* = 14, males, *n* = 10). Values represent the mean ± SEM in each group. *p ≤ .02; ^H^*p* ≤ .1, compared with WT.

The mouse drinking data are presented as mean ± SEM. Alcohol drinking data were analyzed with a repeated measures 2-way analysis of variance with a heterogeneous variance structure to account for difference in variance across ethanol concentration. Degrees of freedom for all tests were estimated with the Kenward-Roger correction. Means and SEMs shown in Figure 1 were calculated with the least squares method. Between-group comparisons were performed with linear contrasts within the repeated measures 2-way analysis of variance. The analyses were carried out with the MIXED procedure in SAS (SAS Institute, Cary, North Carolina). *p* values of < .05 were considered statistically significant.

## Alcohol-Dependent Patients

Seventeen hundred three patients (1349 men, 354 women) meeting criteria of alcohol dependence according to DSM-IV and ICD-10 were recruited from an inpatient abstention program after detoxification at the Department of Psychiatry at Regensburg University. All participants were of German descent, with parents living in Bavaria and participants themselves born and raised in this area. Written informed consent was obtained for all participants before the study. Patients were assessed by trained staff who rated participants independently with a structured interview on the basis of the substance abuse section of the Munich-Composites International Diagnostic Interview (23). Patients with a lifetime history of schizophrenia or an addiction to drugs other than tobacco or alcohol were excluded from the study. Participants were screened for neurological and psychiatric disorders with self-report questionnaires adapted from the German version of the inventory to diagnose depression (24); alcohol consumption levels during the past 30 days were determined with the Alcohol Use Disorders Identification Test (25) or the time-line-follow back (26). Phenotypic description of these patients is displayed in Table 1. Analyses performed in this study are based on 1213 individuals (290 women and 923 men) for whom genotyping data were available (details in the following text).

## Control Subjects

Thirteen hundred forty-seven individuals (714 men, 633 women) from the region of Bonn, Germany, were recruited from 2001 to 2003 within the German National Research Project to serve as control subjects for genetic studies in several neuropsychiatric phenotypes. Population-based recruitment was performed in collaboration with the local census bureau. Participants were screened for neurological and psychiatric disorders with self-report questionnaires adapted from the German version of the inventory to diagnose depression (24,27); smoking and drinking were diagnosed with the Fagerstrom Tolerance Questionnaire (28) and the alcohol use disorder identification test (25). More than 96% of the participants were of German or Western European origin as ascertained by place of birth of their grandparents. Written informed consent was obtained before study participation.

## Single Nucleotide Polymorphism Selection, Genotyping, and Statistical Analysis

Genomic DNA was extracted from whole blood with standard methods and quantified in the Laboratory at the Central Institute of Mental Health Molecular Genetics Laboratory. Seven single nucleotide polymorphisms (SNPs) that captured 74% of HapMap alleles at the *ADCY7* locus on chromosome 16 at *r*^2^ ≥ .8 were selected for genotyping. Single nucleotide polymorphisms were genotyped with the TaqMan MGB biallelic discrimination system. Probes and primers were ordered from and automatically designed by Applied Biosystems (Foster City, California) with the Assay-by-Design product. Polymerase chain reactions were performed in Biometra T1 thermocyclers, and fluorescence was measured with an ABI Prism 7900HT sequence-detector end point read. Genotyping errors and consistency across plates were checked with four common control samples/plate. Process and genotyping data were exported into an internal laboratory information management system. Case-control analyses were conducted to examine allelic association between SNPs and alcohol dependence (categorical variable DSM-IV diagnosis) with PLINK v1.06 (http://pngu.mgh.harvard.edu/∼purcell/plink/) (29). Thresholds for filters were .01 for allele frequency, .1 for missingness/individual, .1 for missingness/marker, and .05 for Hardy–Weinberg equilibrium. Empiric significance levels were obtained after 10,000 permutations.

## Expression Quantitative Trait Loci Analyses in Blood and Adipose Tissues

The correlation between rs2302717 and the level of *ADCY7* messenger RNA (mRNA) was tested in a dataset that includes measurements of the expression of 23,720 transcripts in adipose tissue and in whole blood from 674 and 1002 Icelandic individuals, respectively. The collection of the tissue samples and the measurement of the expression were described previously (30). Of those individuals, 606 with adipose tissue samples and 747 with blood samples had been genotyped with the Illumina 317K or 370K chip, and because rs2302717 is not included on those SNP chips, these genotypes were used to impute genotypes for rs2302717 on the basis of the HapMap CEU (r22) dataset with the IMPUTE program (31). The correlation between *ADCY7* mRNA levels and rs2302717 was tested by regressing the adjusted and inverse normal transformed mean logarithm (log_10_) expression ratio on the number of copies of the risk variant a person carries. For adipose tissue, the expression was adjusted for age and gender of the individuals, and for blood the expression was also adjusted for differential cell count. To adjust for the relatedness of individuals in the expression dataset, the *χ*^2^ statistic was divided by the adjustment factors 1.063 and 1.078 for adipose tissue and blood, respectively. These adjustment factors were determined by simulation on the basis of the known relatedness of the individuals as described previously (32). The gene expression data are available from the GEO database under the accession numbers GSE7965 and GPL3991.

## Results

### Alcohol Preference and Intake by *Adcy7*^+/−^ and WT Mice

We compared ethanol consumption and preference of *Adcy7*^*+/−*^ and WT mice in a two-bottle choice alcohol-drinking paradigm. Differences in alcohol consumption and preference between *Adcy7*^+/−^ animals and their respective WT control subjects, on two genetic backgrounds (C57BL/6 or 129S6/SvEv), are shown in Figure 1. Female WT mice on the C57BL/6 background showed relatively high ethanol preference, in the range of 70%–80%. There was a trend toward an effect of genotype on alcohol preference in these mice [*F*(1,11.3) = 4.39, *p* = .059]. Post hoc linear contrasts showed that preference was significantly higher in the *Adcy7*^+/−^ female mice, compared with WT, at 5% ethanol (*p* = .007), and was also higher at 3% (*p* = .088) and 7% (*p* = .061) ethanol. In male mice on the C57BL/6 background, there was a trend toward a genotype × alcohol concentration interaction with respect to alcohol consumption [*F*(4.235) = 2.2, *p* = .097], but the post hoc comparisons were not statistically significant.

Unaltered female WT mice on the 129S6/SvEv background showed lower alcohol preference (in the range of 20%–40%) than mice on the C57BL/6 background. For these mice, there were significant genotype (deletion of one copy of *Adcy7*) × concentration interactions both for alcohol consumption [*F*(4,34.7) = 5.61, *p* = .0001] and for alcohol preference [*F*(4,33.3) = 5.1, *p* = .003]. The *Adcy7*^+/−^ mice consumed more alcohol at the 10% (*p* = .01) and 14% (*p* = .004) concentrations and also displayed higher preference at these concentrations (10%, *p* = .021; 14%, *p* = .02). For male mice on the 129S6/SvEv background, there was a trend toward a genotype × concentration interaction for alcohol consumption [*F*(4,24.1) = 2.41, *p* = .077], but in juxtaposition to female mice, the *Adcy7*^+/−^ male mice consumed less alcohol than the WT mice at the 14% concentration (*p* = .005).

### Association of *ADCY7* Genotypes with Alcohol Dependence in Humans

We hypothesized that variations in the *ADCY7* gene in humans might also confer susceptibility to differences in alcohol consumption, which could affect the development of dependence. We analyzed association of SNPs within *ADCY7* with alcohol dependence in a sample of 1703 alcohol-dependent patients and 1347 control subjects. Seven haplotype-tagging SNPs were genotyped and tested for association with alcohol dependence. We performed the analysis in males and females separately, given the reported sex-specific role of this gene in humans (22) and our results with the genetically modified mice. The SNP rs2302717 had the most significant, sex-specific association with alcohol dependence (Table 2), and this association remained significant after 10,000 permutations (*p* = .0139 after permutations). Its minor allele (T) was less frequent among alcoholic females than nonalcoholic females. An odds ratio of .71 (95% confidence interval: .54–.93) suggests that the T allele at rs2302717 reduces the risk to develop alcohol dependence by a factor of approximately 1.4 in females. Relative positions of the SNPs genotyped and their linkage disequilibrium (LD) patterns in our sample are shown in Table 3. Haplotype blocks estimation revealed two 2-SNP haplotypes formed by rs2302717/rs7191958 and rs1872691/rs1064448, respectively. Analysis of common haplotypes (frequency ≥ .01) within the rs2302717/rs7191958 block indicated that the minor alleles at these SNPs always co-occurred on the same haplotype (observed frequencies for rs2302717/rs7191958 haplotypes: T/A, .176; C/A, .124; C/G, .696). This indicates that the TA haplotype formed by the combination of rs2302717 and rs7191958 is responsible for the observed associations with alcohol dependence in our sample.

**Figure 2:**
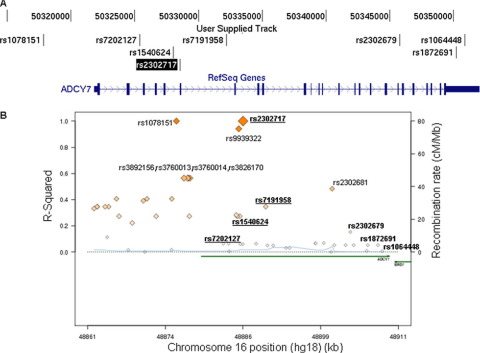
Associations between alcohol dependence in females and single nucleotide polymorphisms in the *ADCY7* locus. **(A)** Genomic organization of the *ADCY7* Reference Sequence (RefSeq) on chromosome 16, generated from University of California at Santa Cruz genome browser with hg19 coordinates. Introns are displayed as thin lines between exons (thick marks). Arrowheads show orientation of the gene on the chromosome. The single nucleotide polymorphisms investigated are indicated, with the marker most significantly associated with dependence (rs2302717) highlighted. A marker (rs1078151) in complete linkage disequilibrium (LD) (*r*^2^ = 1) with rs230271 is also indicated. Upper lane indicates genomic coordinates (base pairs; hg19) on chromosome 16. **(B)** The LD structure around the *ADCY7* locus is shown. Diamonds are colored in a white-to-red scale corresponding to *r*^2^ values from 0 to 1 with rs2302717. Markers genotyped in this study are highlighted in bold; those part of the four single nucleotide polymorphisms-haplotype associated with alcohol dependence are underlined. Estimated recombination rates in **(B)** are from HapMap and gene annotations from University of California at Santa Cruz genome browser with hg18 coordinates.

**Figure 3:**
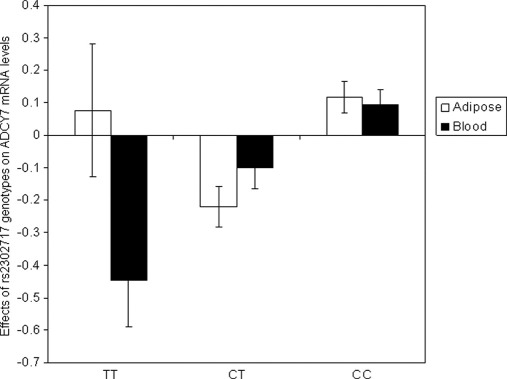
Correlation of rs2302717 genotypes with *ADCY7* (NM_001114) messenger RNA expression in adipose tissue (*n* = 606) and blood (*n* = 747) samples. Data (± SEM) are expressed in standardized (inverse-normal transformed) and adjusted (for sex, age, and differential cell count) expression levels.

The SNP most associated with alcohol dependence (rs2302717) is located in intron five of the *ADCY7* gene and is in high LD with several markers at the 5′ region of the *ADCY7* gene (Figure 2B). Notably, it completely tags rs1078151, which is located 4007 base pairs upstream of the first exon (complete LD [*r*^2^ = 1] between rs2302717 and rs1078151). The location of rs1078151 and its LD with rs2302717 suggested that the minor allele at rs2302717 (and rs1078151) might correlate with changes in *ADCY7* gene expression. To test this, we analyzed whether rs2302717 correlated with *ADCY7* mRNA levels in large population-based blood and adipose tissue cohorts for which genotypes and global gene expression data were available (30). These expression quantitative trait loci analyses revealed that the minor allele at rs2302717 correlated with lower *ADCY7* mRNA levels in both tissues (effect estimates [SEM], *p* value: −.212 [.071], .0039 and −.231 [.068], .001, for adipose tissue and blood, respectively) (Figure 3). These data suggest that genotype at rs2302717 might correlate with *ADCY7* mRNA expression.

## Discussion

Our findings provide the first evidence for a role for *ADCY7* in development of alcohol dependence in women. Although behavioral experiments using genetically modified mice demonstrate a sex-specific role for this gene in voluntary alcohol consumption, the human genetic study identifies a genetic polymorphism within the *ADCY7* gene associating with alcohol dependence in human female subjects. The results obtained in mice support the interpretation that decreased levels of AC7 predispose to higher alcohol intake. This agrees with earlier findings linking decreased activity of the downstream cAMP/PKA/CREB pathway to high voluntary alcohol consumption in rodents. As noted in the preceding text, enhanced voluntary alcohol consumption was found in mice lacking the regulatory IIβ subunit of PKA (11) or lacking CREB (9). Particularly, decreased activity of this pathway in the nucleus accumbens was shown to confer high alcohol drinking. In light of our results, we would now like to extend this suggestion and propose that differences in the expression of AC7, upstream of PKA, modify alcohol-drinking behavior. This hypothesis might be consistent with the fact that AC7 is a selective target of alcohol (33,34), which increases its activity.

The consumption of ethanol is the behavioral act that initiates intoxication (with both positive and negative reinforcing consequences as well as aversive events). It is also the consumption of ethanol in high quantities over periods that results in neuroadaptive changes that lead to signs of alcohol abuse and dependence. Levels of alcohol consumption are statistically correlated with the propensity to become dependent or stay dependent on alcohol (35), and the consumption of ethanol is a necessary (albeit maybe not sufficient) component of predisposition to alcoholism in humans. Given these data and that animal (rodent) models of alcohol dependence do not well-model (although some claims have been made to this effect) the psychiatric criteria (e.g., DSM-IV or ICD-10) of alcohol dependence (except for the hyperexcitability of alcohol withdrawal and tolerance), we currently believe that measuring drinking levels in animals provides a reasonable surrogate of predisposition to alcohol dependence in humans and that a genetic marker that is associated with differences in the quantity of alcohol consumption in animals (including humans) might also be associated with the presence of alcohol dependence in humans.

Previous studies have shown significantly lower receptor-stimulated AC activity in platelets and lymphocytes from abstinent alcoholic subjects compared with nonalcoholic subjects (16,36–38). Because both genetic factors and abstinence from alcohol seem to have roles in determining low platelet AC activity (17,18,38), these data suggested that platelet AC activity could be a biochemical state or trait marker for alcohol dependence. These observations suggested, given that the AC7 isoform is expressed in human platelet precursor cells (39), that AC7 might be associated with development and/or maintenance of alcohol dependence in humans. Prior analysis of AC7 polymorphic repeats in 30 alcoholic and 17 control individuals failed to reveal an association between AC7 variations and alcoholism (40). However, these repeats are in the 3′ untranslated region of *ADCY7*, whereas the haplotype identified in our current work is in the area of the gene transcription start site. Our current study also uses a much larger sample of alcohol-dependent individuals than the prior work (40), to allow robust investigation of *ADCY7* in human alcohol dependence. Our expression quantitative trait loci analyses indicate that the marker most associated with alcohol dependence in our study (rs2302717) and/or other SNPs in LD (like rs1078151) affects *ADCY7* expression. Presence of the minor allele at rs2302717 is associated with lower levels of *ADCY7* mRNA levels in adipose tissue and particularly in blood.

Because lower levels of *ADCY7* mRNA (assuming that mRNA levels quantitatively reflect the levels of AC7 protein) would be expected to be associated with higher alcohol drinking (on the basis of our data with *Adcy7*^+/−^ mice and prior animal studies described in the preceding text), it might seem counterintuitive that the presence of the minor allele at rs2302717 is also associated with a reduced risk of alcohol dependence. Certainly, one should determine the relation of the minor allele at rs2302717 and/or rs1078151 with *ADCY7* mRNA levels in human brain tissue, because genetic variation can affect gene expression in a tissue- and cell-specific manner (41). The importance of tissue type and sampling conditions for the effect of promoter variants on gene expression has been repeatedly observed. A recent example for such effects in various brain regions and blood is provided by rs16147 at the *NPY* locus (42–44). Nongenetic factors might also influence *ADCY7* expression. For example, an explanation of the lower *ADCY7* mRNA levels in humans might well be linked to observations that alcohol intake can lower AC7 enzyme activity in blood (particularly platelets) (17). Both alcohol intake and recent abstinence (withdrawal) have been shown to alter gene expression (45,46). Thus, the relation between rs2302717 and *ADCY7* mRNA might reflect the influence of other factors, including alcohol-drinking history, on gene transcription in individuals of a particular genotype. In other words, the relation we report between the presence of the minor allele of rs2302717 and tissue *ADCY7* mRNA levels might be inherent and/or reflect an interaction with other genetic and nongenetic factors.

It was of interest to note that the female mice of the C57BL/6 strain increased their consumption of ethanol primarily at the lower concentrations, whereas the female 129 mice increased consumption of the higher concentrations of ethanol. The C57BL/6 strain normally prefers the higher concentrations of ethanol (10%) (47), whereas the 129 strain normally shows avoidance of these concentrations (47). In essence, the genetic manipulation (lowering) of AC7 in brain generates an increase in consumption of ethanol at concentrations at which the C57BL/6 mice usually have little interest in the ethanol solution, and 129 mice usually begin to avoid the ethanol solution. At concentrations above 10% ethanol, the C57BL/6 mice are reaching a ceiling with regard to their volume of ethanol solution consumption, and the manipulation of AC7 cannot further increase consumption.

Our observations point to a sex-specific role for AC7, both in mice and humans, with main effects observed in female subjects. Such sex-related differences might not be evident when the cAMP signaling cascade is perturbed at other points. No differences between sexes in measures of ethanol consumption were observed in mice lacking the regulatory IIβ subunit of PKA (11). However, female mutant mice displayed decreased operant self-administration compared with WT females, indicative of decreased reinforcement-seeking behavior (48). Whether CREB deletion contributes to sex-specific effects on alcohol consumption has not been investigated, because only male mice were used for these studies (49). That the CREB-deficient mice were also on a mixed C57BL/6 and 129/SVJ background might also influence the measurements of alcohol consumption levels, compared with males on an isogenic background. Nonetheless, our results reveal a special relevance for AC7 in female alcohol-related behavior.

The sex differences observed in our experiments might not be surprising, considering the signaling crosstalk between the cAMP/PKA pathway and steroid receptors. Recent experiments have demonstrated that elevated levels of cAMP lead to ligand-independent activation of the estrogen receptor through PKA (50,51). Conversely, acute and long-term treatments with estrogens have been shown to increase CREB-DNA binding in brain areas linked to affective and cognitive behaviors, like the amygdala and the frontal cortex (52). In line with this, several studies have reported associations between polymorphisms in the estrogen receptor α gene (*ERS1*) and anxiety in humans (53–55). However, whether such polymorphisms produce differential interaction with components of the cAMP signaling system and also contribute to sex differences in alcohol use and dependence (56) remains to be tested.

Taken together, our results solidify the notion that the cAMP pathway functions in a sex-specific manner to influence alcohol-related behavioral events. We have shown that AC7 is an important AC isoform in mediating the cAMP influence on alcohol-drinking–related behaviors in mice. Our work with humans provides for a sex-specific association of AC7 polymorphisms and alcohol dependence. Further understanding of the regulation of AC7 activity and expression will help provide a better understanding of the AC7-dependent pathways that predispose to alcoholism.

## Figures and Tables

**Table 1:** Phenotypic Characterization of the Alcohol-Dependent Patient Sample

Variables	*n* ± SD
Number of Patients	
All	1703
Women	354 (20.8%)
Men	1349 (79.2%)
Age	
All	43.3 ± 9.9
Women	44.4 ± 10.0
Men	42.9 ± 10.0
Number of Criteria	
ICD-10:	
All	5.4 ± .9
Women	5.3 ± 1.0
Men	5.4 ± .9
DSM-IV:	
All	6.0 ± 1.2
Women	5.0 ± 1.3
Men	6.0 ± 1.2
Age at Begin of Specific Symptoms	
Consumption Alcohol Regularly:	
All	20.8 ± 8.5
Women	25.3 ± 10.6
Men	19.4 ± 7.3
Begin Loss of Control:	
All	28.1 ± 10.6
Women	33.1 ± 10.9
Men	26.7 ± 10.1
Begin Tolerance:	
All	29.5 ± 10.3
Women	34.7 ± 10.2
Men	28.0 ± 9.8
Begin Withdrawal:	
All	34.5 ± 10.3
Women	38.1 ± 10.1
Men	33.4 ± 10.2
First Inpatient Treatment:	
All	39.2 ± 10.3
Women	40.4 ± 10.5
Men	38.8 ± 10.2
Amount of Alcohol Consumption (gm)	
Daily:	
All	206.1 ± 114.1
Women	166.7 ± 96.03
Men	218.1 ± 116.5
“Record” in the last 30 Days Before Inpatient Treatment:	
All	296.2 ± 151.9
Women	226.1 ± 125.1
Men	316.4 ± 153.1
Smoking (investigated *n* = 1248)	
Smokers:	
All	970 (77.7%)
Women	208 (71.7% of women)
Men	762 (79.5% of men)
Number of Cigarettes/Day:	
All	25.9 ± 11.5
Women	23.6 ± 11.3
Men	26.5 ± 11.5

All patients expressed signs of severe alcohol dependence with daily drinking and loss of control.

**Table 2:** Association of *ADCY7* with Alcohol Dependence in Humans

SNP	BP	Minor Allele	MAF in Cases	MAF in Control Subjects	Major Allele	CHISQ	OR (95% CI)	*p*
Females (279 cases, 573 control subjects)								
rs7202127	48,882,871	C	.244	.243	G	.003	1.006 (.795–1.274)	.9588
rs1540624	48,885,507	G	.401	.445	A	2.908	.8364 (.681–1.027)	.0881
rs2302717	48,886,027	T	.149	.198	C	6.137	.7074 (.538–.931)	.0132^a^^b^
rs7191958	48,889,674	A	.267	.316	G	4.266	.789 (.630–.988)	.0389^a^
rs2302679	48,903,279	T	.362	.374	C	.245	.9483 (.769–1.17)	.6206
rs1872691	48,907,711	A	.195	.170	G	1.624	1.184 (.913–1.535)	.2026
rs1064448	48,908,384	T	.475	.464	G	.172	1.044 (.852–1.278)	.6782
Males (1090 cases, 636 control subjects)								
rs7202127	48,882,871	C	.243	.256	G	.661	.936 (.798–1.098)	.4162
rs1540624	48,885,507	G	.430	.443	A	.497	.9511 (.827–1.093)	.4807
rs2302717	48,886,027	T	.184	.179	C	.137	1.034 (.864–1.238)	.7117
rs7191958	48,889,674	A	.301	.314	G	.662	.9398 (.809–1.092)	.4159
rs2302679	48,903,279	T	.374	.351	C	1.815	1.104 (.956–1.275)	.1779
rs1872691	48,907,711	A	.183	.174	G	.425	1.062 (.886–1.273)	.5143
rs1064448	48,908,384	T	.485	.462	G	1.712	1.097 (.955–1.26)	.1907

BP, base pair; CHISQ, Chi-squared test; CI, confidence interval; MAF, minor allele frequency; OR, estimated odds ratio; SNP, single nucleotide polymorphisms.

a Associations attaining nominal significance (*p* ≤ .05).

b Those remaining significant after 10,000 permutations.

**Table 3:** LD Structure in Our Sample

	rs7202127	rs1540624	rs2302717	rs7191958	rs2302679	rs1872691	rs1064448
rs7202127	1	.4	.08	.15	.04	.02	.12
rs1540624	.4	1	.29	.05	.04	.06	.13
rs2302717	.08	.29	1	.48	.24	.03	.01
rs7191958	.15	.05	.48	1	.05	.05	.02
rs2302679	.04	.04	.24	.05	1	.12	.1
rs1872691	.02	.06	.03	.05	.12	1	.23
rs1064448	.12	.13	.01	.02	.1	.23	1

Values represent *r*^2^ values between the indicated pairs of single nucleotide polymorphisms. LD, linkage disequilibrium.
